# Association of Child Maltreatment With Risk of Death During Childhood in South Australia

**DOI:** 10.1001/jamanetworkopen.2021.13221

**Published:** 2021-06-10

**Authors:** Leonie Segal, James Doidge, Jason M. Armfield, Emmanuel S. Gnanamanickam, David B. Preen, Derek S. Brown, Ha Nguyen

**Affiliations:** 1 Health Economics and Social Policy Group, Australian Centre for Precision Health, University of South Australia, Adelaide, South Australia, Australia; 2 Intensive Care National Audit & Research Centre, London, United Kingdom; 3 School of Population and Global Health, University of Western Australia, Perth, Western Australia, Australia; 4 Brown School, Institute for Public Health, Washington University in St Louis, St Louis, Missouri

## Abstract

**Importance:**

Child maltreatment is a prominent public health concern affecting 20% to 50% of children worldwide. Consequences for mental and physical health have been reported, but population-level estimates of risk of death during childhood that are adjusted for confounders have not been published to date.

**Objective:**

To estimate the association of documented child protection concerns regarding maltreatment with risk of death from infancy to 16 years of age.

**Design, Setting, and Participants:**

This case-control study was nested in a population birth cohort of 608 547 persons born in South Australia, Australia. Case children were those who died between 1 month and 16 years of age (with the death registered by May 31, 2019). Control children were randomly selected individuals from the same population who were alive at the age at which the case child died, matched 5:1 for age, sex, and Aboriginal status. Data were analyzed from January 2019 to March 2021.

**Exposure:**

Children were assigned to 1 of 4 child protection concern categories (child protection system notification[s] only, investigation[s] [not substantiated], substantiated maltreatment, and ever placed in out-of-home care) based on administrative data from the South Australia Department for Child Protection or were classified as unexposed.

**Main Outcomes and Measures:**

Mortality rate ratios for death before 16 years of age, by child protection concern category, were estimated using conditional logistic regression, adjusted for birth outcomes, maternal attributes, and area-based socioeconomic status. Patterns of cause of death were compared for children with vs without child protection concerns.

**Results:**

Of 606 665 children included in the study, 1635 were case children (57.9% male [when sex was known]; mean [SD] age, 3.59 [4.56] years) and 8175 were control children (57.7% male; mean [SD] age, 3.59 [4.56] years [age censored at the time of death of the matched control child]). Compared with children with no child protection system contact, adjusted mortality rate ratios among children who died before 16 years of age were 2.69 (95% CI, 2.05-3.54) for children with child protection system notification(s) only; 3.16 (95% CI, 2.25-4.43) for children with investigation(s) (not substantiated); 2.93 (95% CI, 1.95-4.40) with substantiated maltreatment; and 3.79 (95% CI, 2.46-5.85) for children ever placed in out-of-home care. External causes represented 136 of 314 deaths (43.3%) among children with a documented child protection concern and 288 of 1306 deaths (22.1%) among other children. Deaths from assault or self-harm were most overrepresented, accounting for 11.1% of deaths in children with child protection concerns but just 0.8% of deaths among other children.

**Conclusions and Relevance:**

In this case-control study, children with documented child protection concerns, who were known to child protection agencies and were typically seen by clinicians and other service providers, had a higher risk of death compared with children with no child protection service contact. These findings suggest the need for a more comprehensive service response for children with protection concerns.

## Key Points

**Question:**

What is the differential risk of death before age 16 years among children with documented child protection concerns compared with children with no child protection system contact in South Australia?

**Findings:**

In this case-control study of 606 665 children in Australia, those with documented child protection concerns had greater adjusted mortality rate ratios before 16 years of age compared with children with no child protection system contact after adjusting for child and maternal factors and socioeconomic status. These results varied by child protection system category.

**Meaning:**

These findings suggest the need for a more comprehensive service response for children with protection concerns.

## Teaser

This case-control study assesses the association of documented child protection concerns regarding child maltreatment with risk of death from infancy to 16 years of age in South Australia.

## Introduction

Child abuse and neglect are prominent worldwide public health problems.^1,2^ Rates vary by region and type of abuse; physical and emotional abuse are estimated to affect more than 35% of children in Asia, more than 50% of children in Africa and South America, 30% in North America, and 12.5% of children in Europe and Australia.^2^ A reported 15% to 50% of children may be exposed to neglect^2^; thus, child maltreatment, which covers abuse and neglect, affects approximately 20% to 50% of children worldwide.

Child maltreatment can start in utero (eg, with harmful drug use by the mother) but occurs throughout childhood.^3^ The consequences of child maltreatment are considerable and can be lifelong.^4,5^ Associations of child maltreatment with mental and physical health, including with increased risk for attempted (and completed) suicide,^6,7,8^ mental illness, and addiction disorders^8,9^ and higher rates of hospitalization,^10^ have been widely documented.

The mechanisms underpinning the health consequences of child maltreatment are well described.^11,12,13,14^ In children, the association of child maltreatment with injury, illness, and potential death is most direct in cases of serious physical or sexual abuse or profound neglect (supervisory, medical, and basic care). In addition, child maltreatment is associated with changes in the developing brain,^11,12^ characterized as toxic stress,^13^ affecting cognition, emotions, behaviors, and relational modeling,^14^ which may be associated with additional harm.

The association of death with child abuse and neglect has been studied primarily through detailed case reviews.^15,16,17,18^ Statutory review committees on death during childhood have been established in many jurisdictions to examine the circumstances of deaths among children to inform strategies to reduce potentially avoidable deaths.^19,20,21^ A recent study by such a committee revealed that deaths among children with family-level child protection system (CPS) involvement (child, sibling, or parent) were associated with a substantially higher proportion of deaths coded as “undetermined/sudden infant death” or “external cause” than deaths with no CPS connection.^20^ However, these studies were not population based and thus could not establish population-level risk estimates.

A review of studies of deaths among children and child maltreatment concluded that “the true incidence of fatal child abuse and neglect is unknown.”^15^^(p265)^ A literature search revealed only 1 population-based study from 1992 in the US^22^ involving risk of risk of death during childhood (into adolescence) by child maltreatment exposure. The authors reported 2.9 times the risk of death and 20 times the risk of assault (to <18 years of age) among children after a CPS notification compared with a matched comparison group with no notification.^22^ Study estimates were not adjusted for potential confounders (such as birth outcome or socioeconomic status), nor did the authors explore risk by level of child protection concern. A 2011 study by Putnam-Hornstein^23^ that used a similar method estimated the risk of injury death before age 5 years, reporting 5.85 times the risk for intentional and 2 times the risk for deaths from unintentional injury among children after a CPS report compared with children with no report. A 2021 study^24^ on the risk of death from suicide after CPS contact during adolescence that used a nested case-control design reported odds ratios of 3.07 to 5.16 depending on the CPS category.

Evidence of the association of child protection concerns with all-cause death rates throughout childhood is limited. To address this evidence gap, we designed a study to estimate the association between different levels of child maltreatment concern and risk of death among children between 1 month and 16 years of age, adjusting for confounders present at birth. The age of 16 years was the chosen cutoff point, with the observation that deaths from age 16 to 18 years are more aligned with young adulthood, as reported in a recent study.^8^

## Methods

### Study Design

This case-control study, nested within a population cohort of 608 547 children born in the state of South Australia, Australia, between January 1, 1986, and June 30, 2017, included 606 665 children who survived to 1 month of age. The cohort was constructed from linked, deidentified data extracted from the South Australia Birth Registry, the South Australia Perinatal Statistics Collection, and the South Australia Death Registry. Case children were defined as all children who died in the cohort before 16 years of age with the death registered by May 31, 2019. Control children were randomly selected with replacement in a 5:1 ratio from the population of children alive at the time of the death of the case child and matched for month and year of birth, sex, and Aboriginal status (as described in administrative data^3^ and included as a match variable, noting higher death rates among Aboriginal children compared with non-Aboriginal children^25^). Ethical approval for the study was obtained from the South Australia Department for Health and Wellbeing Human Research Ethics Committee and the University of South Australia. The South Australia Department for Health and Wellbeing Human Research Ethics Committee determined that individual informed consent was not required because the study used deidentified historic administrative data. This study followed the Strengthening the Reporting of Observational Studies in Epidemiology (STROBE) reporting guideline.

The nested case-control design was adopted as the most rigorous study design to address our research objective to compare childhood mortality rates across CPS contact categories with consideration of the time-varying nature of CPS contact. This design allowed us to directly compare mortality rate ratios (MRRs) across CPS categories using a single, age-matched population sample while addressing immortal time bias and allowing for the complex transition pathways between CPS categories throughout childhood, thereby making the best use of the available population cohort data. Data linkage was undertaken by SA-NT DataLink,^26^ the accredited data linkage agency for South Australia, using best practice determinist and probabilistic linkage. Extensive clerical review was performed, drawing on more than 50 data sets to deliver an estimated 99.6% accuracy (0.4% false-positive rate) and a 0.8% false-negative or missed links rate.^27^ The research team received the deidentified data from the data custodians, with encrypted, project-specific linkage keys to enable merging across data sets.

### Exposure Definitions

Documented child protection concern was defined by CPS involvement, determined from the South Australia Department for Child Protection administrative data files (January 1, 1986, to June 30, 2017). Five mutually exclusive and graded child protection concern categories were developed reflecting Department of Child Protection risk assessment^28^: (1) no CPS contact, the reference condition indicating no or low risk of child maltreatment; (2) notification recorded but no investigation (reflecting insufficient information, failure to meet child maltreatment concern thresholds, threat not current, or insufficient agency resources for follow-up); (3) investigation but no substantiation; (4) recorded, substantiated abuse or neglect but no placement in out-of-home care (OOHC); and (5) at least 1 placement in OOHC, indicating extreme risk. For control children, CPS exposure status was based on their child protection history to age at death of the matched case child to account for potential immortal time bias.

### Outcomes

Occurrence of death and month and year of death were identified from the South Australia Death Registry and used to identify study cases and children still alive at 1 month, augmented by perinatal records. Deaths before 1 month were excluded from the study because complications of pregnancy, birth trauma, and congenital defects are primarily responsible for perinatal deaths and owing to the minimal opportunity for CPS contact.

Information on cause of death was provided through the National Codified Cause of Death Repository and the National Coronial Information System for deaths between 2006 and 2019 and through a historic coded data set available through the SA-NT DataLink for deaths to 2006. Data capture was equivalent, and coding for cause of death in both data sets was completed by the Australian Bureau of Statistics, the designated national authority for this task. For the 189 case children (12%) for whom only cause-of-death text fields were reported, coding was completed independently by 2 individuals (including H.N.) according to Australian Bureau of Statistics coding criteria and was reviewed by another one of us (L.S.).

Deaths were allocated using *International Statistical Classification of Diseases and Related Health Problems, Tenth Revision (ICD-10)*^29^ or *International Classification of Diseases, Ninth Revision* (*ICD-9*) codes^30^ to natural causes (chapters I-XVIII) or external causes. Deaths from natural causes related to the perinatal period (*ICD-10* codes P00-P96 or *ICD-9* codes 760-779) (eg, congenital malformations [*ICD-10* codes Q00-Q99 or *ICD-9* codes 740-759], undetermined or consistent with sudden infant death syndrome [*ICD-10* codes R95-R99 or *ICD-9* codes 798-799]) were identified separately. Deaths from external causes (chapters XIX and XX) were further classified (assault [*ICD-10* codes X85-Y09 or *ICD-9* codes E960-E969]; self-harm [*ICD-10* codes X60-X84 or *ICD-9* codes E950-E959]; transport related [*ICD-10* codes V01-V99 or *ICD-9* codes E800-E807, E10-E819, E820-E838]; drownings [*ICD-10* codes W65-W74 or ICD-9 codes E910, E984]; suffocation, strangulation, or asphyxia [*ICD-10* codes T71, W75, W76, X88-X89 or *ICD-9* codes E911-E913, E915, E953]; fire [*ICD-10* codes T20-T32, X00-X09 or *ICD-9* codes E890-E899]; falls [*ICD-10* codes W00-W19 or *ICD-9* codes E880-E888]; poisoning [*ICD-10* codes T36-T50, X40-X41 or *ICD-9* codes E980-E989]; and suspicious death [possible self-harm, assault, or accidental death (*ICD-10* codes Y10-Y34 or *ICD-9* codes E960-E969 and E980-E989)]).

### Covariates

A set of covariates was selected for inclusion in the multivariable analysis from established risk factors for death during childhood^31^ or factors that might moderate the association between child maltreatment and death and included 4 birth outcomes sourced from the perinatal data: child still in hospital at 28 days after birth (yes or no), congenital abnormality (yes or no), preterm birth before 37 weeks’ gestation (yes or no), and low birth weight (<2500 g) (yes or no). Also included were maternal attributes at the time of the child’s birth sourced from the birth and perinatal data: age (<21 years or ≥21 years), marital status (married or in a de facto relationship, or neither), employment status (employed or not employed), and maternal smoking during pregnancy (yes or no). Area-based socioeconomic status using suburb and postal code of the mother, sourced from birth records, was mapped against the Index of Relative Socioeconomic Disadvantage^32^ and classified as quintiles using Australian cutoff points. Child sex was not included as a covariate because in a nested case control design using conditional logistic regression, match variables cannot be included in the analysis.

### Statistical Analysis

Key characteristics of case and control children are described using descriptive statistics. Percentages of deaths by category were ascertained, and percentages of deaths from natural causes and external causes (and the aforementioned subcategories) were compared between children with and without CPS contact.

Associations between CPS involvement and death before 16 years of age, given survival at 1 month, were estimated using conditional logistic regression. Because this was a nested case-control study with incidence density sampling, the odds ratios produced by logistic regression provided consistent estimators of MRRs for each CPS group compared with no CPS group. Separate models were fit with and without adjustment for the listed covariates. Unadjusted and adjusted MRRs were reported with 95% CIs and 2-sided *P* values. Statistical significance was set at *P* < .05. Separate models were run for male and female children with a dichotomized exposure of any CPS contact vs none. All analyses were conducted using Stata, version 16.0 (StataCorp LLC).

## Results

### Descriptive Analysis

Of 606 665 children included in the study, 1635 were case children (57.9% male [when sex was known]; mean (SD) age, 3.59 [4.56] years) and 8175 were control children (57.7% male; mean [SD] age, 3.59 [4.56] years). Overall, 1635 deaths (2.7 per 1000 children) were recorded before 16 years of age by May 31, 2019. Children with unfavorable birth outcomes were more likely to have died (case children) than to survive (control children) (Table 1). Children of mothers who were not in a married or de facto relationship (at the time of the child’s birth), children of younger mothers (age <21 years), and children of mothers in the 3 lower quintiles of socioeconomic status were more likely to have died by 16 years of age.

**Table 1.** Characteristics of Case and Control Children and Their Mothers at the Time of Birth

Characteristic	Children or mothers, No. (%)		*P* value^a^
	Case children (n = 1635)	Control children (n = 8175)	
**Children**			
Age, mean (SD), y	3.59 (4.56)	3.59 (4.56)	NA
Sex			
Male	922 (56.4)	4718 (57.7)	NA
Female	671 (41.0)	3457 (42.3)	
Unknown	42 (2.6)	0	
Birth outcomes			
Still in hospital 28 d after birth	332 (20.3)	157 (1.9)	<.001
Preterm birth	375 (22.9)	593 (7.3)	<.001
Low birth weight	375 (22.9)	514 (6.3)	<.001
Congenital abnormality	278 (17.0)	171 (2.1)	<.001
**Mothers**			
Age, y			
<21	227 (13.9)	679 (8.3)	<.001
≥21	1364 (83.4)	7453 (91.2)	
Unknown	44 (2.7)	43 (0.5)	
Marital status			
Married or de facto relationship	1247 (76.3)	6894 (84.3)	<.001
Not married	373 (22.8)	1205 (14.7)	
Unknown	15 (0.9)	76 (0.9)	
Employment status			
Employed	848 (51.9)	5330 (65.2)	<.001
Not employed	714 (43.7)	2623 (32.1)	
Unknown	73 (4.5)	222 (2.7)	
SES disadvantage^b^			
Quintile 1	601 (38.7)	2520 (31.6)	<.001
Quintiles 2-3	604 (38.9)	2974 (37.2)	
Quintiles 4-5	349 (21.3)	2492 (30.5)	

Abbreviations: NA, not applicable; SES, socioeconomic status.

^a^ *P* values are from the χ^2^ test (case children vs control children).

^b^ Quintile 1 was most disadvantaged, and quintiles 4 to 5 were least disadvantaged.

Case children were more likely to have had CPS contact than were control children; 321 case children (19.6%) had any CPS contact compared with 581 control children (7.1%) (Table 2). Of the 55 deaths among case children who had ever been placed in OOHC, most of these deaths (40 [73%]) occurred when the child was not in care.

**Table 2.** Child Protection System Contact in the Case and Control Groups

CPS involvement^a^	Children, No. (%)	
	Case group	Control group
None	1314 (80.4)	7594 (92.9)
Any	321 (19.6)	581 (7.1)
Notification only	123 (7.5)	269 (3.3)
Investigation, no substantiation	88 (5.4)	145 (1.8)
Substantiation, no OOHC	55 (3.4)	86 (1.1)
Ever OOHC	55 (3.4)^b^	81 (1.0)
Total	1635 (100)	8175 (100)

Abbreviations: CPS, child protection system; OOHC, out-of-home care.

^a^ Mutually exclusive categories as defined in the text.

^b^ Of the 55 case children, 15 died while in OOHC.

The percentage of deaths classified as having an external cause was, among children with any CPS contact, nearly 2 times that of children with no CPS contact at 136 of 314 (43.3%) and 288 of 1306 (22.1%), respectively. Intentional death (or high suspicion of such) accounted for 41 of 314 deaths (13.1%) among children with CPS contact but only 17 of 1306 deaths (1.3%) among children with no CPS contact (Table 3). Considering only deaths coded as suicide or assault, these deaths made up 35 of the 314 deaths (11.1%) of children with any CPS contact and 11 of the 1306 deaths (0.8%) among children with no CPS contact. Only 2 of the 1635 deaths listed a child maltreatment code as a contributing cause of death.

**Table 3.** Cause of Death by Child Protection System Contact Status^a^

Cause of death	Deaths among children, No. (%)		Age, median (IQR), y
	Any CPS contact	No CPS contact	
Natural			
All (chapters I-XIX)^b^	178 (56.7)	1018 (77.9)	NA
Chapters I-XV^c^	113 (36.0)	493 (37.7)	3.06 (0.75-8.67)
Perinatal	7 (2.2)	95 (7.3)	0.17 (0.11-0.43)
Congenital malformation	27 (8.6)	179 (13.7)	0.61 (0.26-2.91)
Undetermined or consistent with SIDS	31 (9.9)	251 (19.2)	0.45 (0.36-2.36)
External			
All (chapters XIX, XX)^d^	136 (43.3)	288 (22.1)	NA
Assault	23 (7.3)	9 (0.7)	2.19 (0.62-4.13)
Self-harm	12 (3.8)	4 (0.3)	15.08 (14.54-15.68)
Suspicious death^e^	6 (1.9)	4 (0.3)	2.27 (0.95-13.43)
Transport related	42 (13.4)	93 (7.1)	7.04 (3.25-11.50)
Drowning	13 (4.1)	56 (4.3)	1.96 (1.41.3.44)
Suffocation, strangulation, or asphyxia	15 (4.8)	52 (4.0)	1.13 (0.46-2.63)
Fire	7 (2.2)	24 (1.8)	3.32 (2.60-5.56)
Fall	6 (1.9)	5 (0.4)	6.03 (1.94-9.94)
Poisoning	3 (1.0)	10 (0.8)	3.93 (2.28-11.56)
Other	9 (2.9)	31 (2.4)	3.08 (0.97-7.27)
Total^f^	314 (100)	1306 (100)	NA

Abbreviations: CPS, child protection system; *ICD-10*, *International Statistical Classification of Diseases and Related Health Problems, Tenth Revision*; *ICD-9*, *International Classification of Diseases, Ninth Revision*; IQR, interquartile range; NA, not applicable; SIDS, sudden infant death syndrome.

^a^ Cause of death based on *ICD-9* codes^29^ and *ICD-9* codes.^30^

^b^ From chapters I to XVIII of *ICD-10*.

^c^ Chapters I to XV of *ICD-10* cover infectious and parasitic diseases; neoplasms; diseases of the blood and blood-forming organs and involving the immune mechanism; endocrine, nutritional, and metabolic diseases; circulatory system; mental and behavioral disorders; nervous system; the eye; the ear; respiratory system; digestive system; the skin; musculoskeletal system; genitourinary system; and pregnancy and childbirth.

^d^ Codes are given in the Outcomes subsection of the Methods section.

^e^ Possible self-harm, assault, or accidental death or intent unclear: *ICD-10* codes Y10-Y34 or *ICD-9* codes E960-E969 and E980-E989.

^f^ Excluding 32 deaths with missing information on cause of death.

### Multivariable Analysis

Among children in all CPS contact categories, MRRs for death before 16 years of age, given survival to 1 month, were significantly higher than those among children with no CPS contact in the unadjusted and adjusted analyses (Table 4). Mortality rate ratios were generally higher for CPS contact categories, suggesting more serious child protection concerns (although with overlapping 95% CIs). Unadjusted MRRs, which represent the observed risk of child death among children with documented child protection concerns, was between 3.33 (95% CI, 2.62-4.25) and 5.07 (95% CI, 3.52-7.29) across the CPS groupings.

**Table 4.** Univariable and Multivariable Conditional Logistic Regression Results: CPS Contact and Mortality Before 16 Years

Variable	Unadjusted analysis^a^		Adjusted analysis^b^	
	MRR (95% CI)^c^	*P* value	aMRR (95% CI)^c^	*P* value
**CPS involvement**				
None	1 [Reference]	NA	1 [Reference]	NA
Notification only	3.33 (2.62-4.25)	<.001	2.69 (2.05-3.54)	<.001
Investigation, no substantiation	4.30 (3.23-5.71)	<.001	3.16 (2.25-4.43)	<.001
Substantiation, no OOHC	4.53 (3.17-6.48)	.004	2.93 (1.95-4.40)	<.001
Ever OOHC	5.07 (3.52-7.29)	<.001	3.79 (2.46-5.85)	<.001
Any				
All children	4.01 (3.39-4.75)	<.001	2.99 (2.45-3.64)	<.001
Male children	4.29 (3.40-5.41)	<.001	3.41 (2.62-4.45)	<.001
Female children	3.44 (2.67-4.43)	<.001	2.51 (1.85-3.40)	<.001
**Childbirth outcomes**				
In hospital at 28 d vs discharged	12.71 (10.33-15.63)	<.001	5.29 (3.91-7.17)	<.001
Congenital abnormality: yes vs no	9.53 (7.76-11.70)	<.001	6.17 (4.82-7.88)	<.001
Preterm birth: yes vs no	3.81 (3.30-4.40)	<.001	1.27 (0.98-1.64)	.07
Low birth weight: yes vs no	4.50 (3.87-5.23)	<.001	1.42 (1.09-1.84)	.03
**Characteristics of mothers at the time of childbirth**				
Age <21 vs ≥21 y	1.90 (1.61-2.24)	<.001	1.42 (1.16-1.73)	.001
Not married vs married	1.75 (1.53-2.00)	<.001	1.21 (1.02-1.43)	.03
Not employed vs employed	1.90 (1.69-2.15)	<.001	1.36 (1.18-1.58)	.001
Smoking: yes vs no	2.05 (1.73-2.43)	<.001	1.33 (1.08-1.63)	.04
SES disadvantage^d^				
Quintiles 4-5	1 [Reference]	NA	1 [Reference]	NA
Quintiles 2-3 vs quintiles 4-5	1.46 (1.26-1.68)	<.001	1.33 (1.17-1.62)	<.001
Quintile 1 vs quintiles 4-5	1.73 (1.49-2.00)	<.001	1.40 (1.18-1.60)	.002

Abbreviations: aMRR, adjusted mortality rate ratio; CPS, child protection system; MRR, mortality rate ratio; NA, not applicable; OOHC, out-of-home care; SES, socioeconomic status.

^a^ Univariable analysis.

^b^ Multivariable analysis.

^c^ Because of the age-matched, nested case-control design, odds ratios estimated using conditional logistic regression are directly interpretable as MRRs as would be obtained through survival analysis of the population cohort.

^d^ Quintile 1 was most disadvantaged, and quintiles 4 to 5 were least disadvantaged.

Among children who had 1 or more notifications but no investigation, the adjusted MRR of death before age 16 years was 2.69 (95% CI, 2.05-3.54). Among children who had been the subject of an investigation, whether substantiated or not, the adjusted MRRs were similar at 2.93 (95% CI, 1.95-4.40) and 3.16 (95% CI, 2.25-4.43), respectively. Among children who had ever been placed in OOHC, indicating an extreme child protection concern, the adjusted MRR was 3.79 (95% CI, 2.46-5.85). Adjusted MRRs among children with CPS contact were higher for male children (3.41; 95% CI, 2.62-4.45) than for female children (2.51; 95% CI, 1.85 to 3.40). Two birth outcomes were associated with death before age 16 years: still in hospital at 28 days (adjusted MRR, 5.29; 95% CI, 3.91-7.17) and congenital abnormality (adjusted MRR, 6.17; 95% CI, 4.82-7.88). Socioeconomic status and maternal variables, although significant, had by comparison reduced association with risk of death (adjusted MRRs from 1.21; 95% CI, 1.02-1.43 for marital status to 1.42; 95% CI, 1.16-1.73 for young age) after adjustment for other covariates.

Death data were incomplete before 1990. Therefore, as a check, we reran the analysis limiting the cohort to those born from 1990 onward. The MRR for children with any CPS contact vs no CPS contact was unchanged at 2.94 (95% CI, 2.38-3.63) vs 2.99 (95% CI, 2.45-3.64).

## Discussion

In this population-based case-control study, which used data for all deaths from infancy to 16 years of age in a 30-year South Australian birth cohort, incorporated different CPS contact levels, and adjusted for pertinent covariates at birth, an increased risk of death was found among children with documented child protection concerns compared with children with no CPS contact. Unadjusted MRRs, which represent the observed risk of death among children with documented child protection concerns, was between 3.33 (95% CI, 2.62-4.25) and 5.07 (95% CI, 3.52-7.29) across the CPS groupings. After adjusting for birth outcomes, maternal attributes, and socioeconomic status, MRRs among children with documented child protection concerns remained high. The MRRs should be interpreted as an average over the whole observation window of the study because variation throughout childhood is possible.

Two birth outcomes had the strongest association with excess risk of death: still in hospital at 28 days (adjusted MRR, 5.29; 95% CI, 3.91-7.17) and congenital abnormality (adjusted MRR, 6.17; 95% CI, 4.82-7.88). Socioeconomic status and maternal variables were also associated with risk of death (adjusted MRRs, 1.21 [95% CI, 1.02-1.43] to 1.42 [95% CI, 1.16-1.73]) after adjusting for the CPS and birth outcomes. This finding is consistent with results of other studies^8,33^ and underscores the desirability of including a measure of child maltreatment exposure when exploring the association between socioeconomic status and health.^33^ Because an association between child maltreatment and socioeconomic status has been observed,^34^ if child maltreatment is not included in multivariable analysis, a missing variable error is likely to occur as well as a flawed understanding of the factors associated with poor health. If child abuse and neglect and its intergenerational transmission^35^ as well as socioeconomic factors are associated with poor health, the policy implications would be different. Further exploration of this question appears to be needed, including the role of government-funded income, health, education, and other social supports.

Poor birth outcomes, which were significantly associated with early death in our study, are not necessarily confounders. They may be associated with unsafe behaviors during pregnancy, such as heavy drinking or illicit drug use (representing neglect of the unborn baby).^36^ As such, the multivariable estimates may be overadjusted, underestimating the association of child protection concerns with risk of death during childhood.

Children who had 1 or more notifications, even when a notification was not escalated to an investigation, had a substantially higher adjusted MRR compared with children with no CPS contact. A possible explanation for this finding is that the opportunity for CPS contact escalation was curtailed by the censoring of CPS contact because of death. A modest further increase in risk was observed for children who underwent investigation (substantiated or not), with further risk escalation among children who had been removed to OOHC, for whom child protection concern was highest.

The risk estimates observed were somewhat higher than estimates from a previous population-based study by Sabotta and Davis^22^ of child maltreatment and child death into adolescence. They reported a 2.9 times unadjusted risk of death for children after a first notification to the CPS compared with children with no CPS contact, and Putnam-Hornstein^23^ reported a 2.59 adjusted MRR for all injury-related deaths before 5 years of age.

In the present study, external causes contributed a higher proportion of deaths among children with CPS contact than did natural causes, and the highest differential proportion was from intentional injury. The percentages of deaths by assault or suicide were low among children without CPS contact (<1%) but accounted for 11.2% of deaths among children with CPS exposure. These findings were consistent with those of Sabotta and Davis,^22^ who reported 18 times the risk of death from homicide among children with prior CPS contact (compared with those who had no CPS contact),^22^ and those of Putnam-Hornstein,^23^ who reported approximately 6 times the risk of death from intentional injury.

As this research was being conducted, the coding of child deaths was found to be incomplete, with family circumstances rarely captured. Only 2 of 1635 deaths listed any child maltreatment as contributing causes. Findings from coroners’ inquests^37^ and case study reports on child deaths^20^ suggest that coded cause of death underestimates child maltreatment as a contributing cause of death during childhood in South Australia. This scenario may also be an issue elsewhere. Ensuring that the family circumstances surrounding a child’s death are recorded as coded cause of death is important because deidentified administrative data are increasingly used to inform policy and practice.

The present study used CPS contact as the measure of child protection concern indicating possible child maltreatment exposure. Alternative case ascertainment through surveys of parents (of their abusive or neglectful behavior toward their infants and children), of teenagers (of their child abuse or neglect history), or of human services professionals (including clinicians) drawing on their observations to ascertain child maltreatment exposure was not a feasible option. Large sample sizes would be required to deliver the power to detect differences in death rates across child maltreatment exposure categories given the low occurrence of deaths among children. In addition, survey data have a number of possible sources of bias including cultural norms, social acceptability pressures, and failure to recollect early-in-life abusive or neglectful events and circumstances. Persons experiencing the worst child maltreatment outcomes are likely underrepresented in population-based surveys.

Using CPS contact as a measure of child protection concern allowed the use of large-scale administrative data. Although some children with child maltreatment exposure may not have had contact with the CPS and some children with CPS contact may not have been exposed to child maltreatment, at the group level, the CPS appeared to have sufficient discrimination to explore the association of interest. In South Australia, individuals with a wide range of occupations who interact with children are legally required to notify the Department for Child Protection of any child protection concerns, reducing the likelihood of ongoing child maltreatment remaining undetected and unreported. Any error in assignment would tend to bias toward the null, such that the large effect estimates observed are potentially underestimates of the true association.

The findings of this study suggest that children with documented child protection concerns have higher rates of death during childhood, especially deaths from injury, suggesting opportunities for prevention. Jurisdictions in many countries have child protection agencies tasked with identifying and protecting children at risk of maltreatment. The findings of this study bring into question the adequacy of current support that these children and their distressed families receive.

Children at risk of maltreatment are coming to the attention of child protection services, clinicians, health services providers, and other agencies. Some programs (eg, For Baby’s Sake, Attachment and Biobehavioral Catch-up, Circle of Security, Parallel Parent and Child Therapy, Incredible Years, and Parents Under Pressure) have been developed to provide intensive therapy-based support for parents (and parents to be) to create a more nurturing environment for their child, with some programs commencing in pregnancy and continuing through middle childhood.^38,39,40,41^ However, many distressed families do not have access to the intensive interdisciplinary and intersectoral family support programs and high-quality therapeutic services that they need. Studies^42,43^ have reported inadequate capacity to deliver the necessary trauma-based therapy to distressed infants, children, and their families. Clinicians and the wider human services workforce require high-level skills, expert supervision, and the opportunity to work in an interdisciplinary setting to successfully meet the needs of infants, children, and their families experiencing extreme adversity.^44^ That outcomes for male individuals were worse than for female individuals in our study warrants further exploration, with possible policy and practice implications.

Researchers have demonstrated that investing in the early childhood years, especially for families with greater disadvantages, can offer a high return on investment in terms of health, education, and productivity.^45^ Especially in a climate of increased stresses on families, as during the COVID-19 global pandemic and with increasing child protection concerns, society should be alert to the situation of children at risk of maltreatment and their distressed families and provide the support needed. Substantial progress has been made over many decades in reducing deaths among children. However, there are still children who do not grow up in a safe and nurturing household, representing a clear opportunity for preventing future deaths during childhood.

### Strengths and Limitations

This study has strengths. Access to linked administrative data are a considerable strength of the study, providing a sufficiently large cohort to explore deaths during childhood and access to information on a range of attributes that were potential confounders, derived at the time of the child’s birth and avoiding the use of covariates collected later in life and potentially associated with child maltreatment exposure.

This study also has limitations. Death ascertainment before 1990 was incomplete. The study used the birth cohort from 1986 to maximize the number of deaths for analysis, with the observation that there was a less than 4 in 10 000 chance that a child selected as a control may have died before 1990. As a check, we reran the analysis from 1990, and the MRR for children with any CPS contact vs no CPS contact was unchanged at 2.94 (95% CI, 2.38-3.63) vs 2.99 (95% CI, 2.45-3.64).

Deaths were limited to those recorded in the South Australia Death Registry, with CPS contact also limited to the South Australia Department of Child Protection; thus, both the exposure and outcome excluded events outside South Australia. Given the case-control study design, it is unclear whether this limitation would have introduced any bias in our estimated MRRs. We had access to partial OOHC data before January 1, 1990. Out-of-home care data included all children who entered care after January 1, 1990, or entered care after January 1, 1986, and were in care on January 1, 1990.

For children who had entered OOHC, the observed MRR reflected the combined association of the child protection concern that triggered the removal, the removal itself, and possible safety concerns related to the alternate placement. Only 15 of 55 deaths among children ever removed occurred while children were in care, but further exploration of this group is needed. Cohorts larger than that in our study are needed to examine the association of child maltreatment and OOHC with risk of death among children to explore age at first (and subsequent) entry to care, care type (foster, kinship, or residential), number of placement changes, total time in care, and child maltreatment exposure characteristics.

## Conclusions

The findings of this study suggest that children with reported child protection concerns have greater risk for death during childhood compared with children with no CPS contact. The high proportion of deaths from external causes suggests an opportunity for prevention.
